# Cross-Species Sex Identification and Comparative Analysis of the *SRY* Gene in American Mammals

**DOI:** 10.3390/ani16131949

**Published:** 2026-06-23

**Authors:** Xinqiu Li, Wei Li, Ningning Wu, Zheng Wang, Yuanyuan Zhang, Ruohui Deng, Chao Du, Huaiyong Mu, Nan Ding, Simin Jiao, Yunyun Zhu, Ruijie Jiang, Zhe Xu, Yongteng Huo, Feier Hao, Chao Bai, Yuyan You

**Affiliations:** Beijing Key Laboratory of Captive Wildlife Technologies, Beijing Zoo, Beijing 100044, China; lwzyj624@sohu.com (W.L.); 15117931258@163.com (N.W.); wangzheng_1980@126.com (Z.W.); zxxbest@sina.com (Y.Z.); dengruohui1993@163.com (R.D.); 13716896127@163.com (C.D.); liuyue1125@sohu.com (H.M.); dingnan34@icloud.com (N.D.); mohukun_jiu@sina.com (S.J.); vetzhuyy@163.com (Y.Z.); jiang6494@alu.cau.edu.cn (R.J.); xuzhe67@sina.com (Z.X.); huoyongteng@126.com (Y.H.); zgdwybb@163.com (F.H.); baichao37@126.com (C.B.)

**Keywords:** *SRY*, cross-species, sex identification, sequence alignment, selective pressure

## Abstract

In zoo husbandry and wildlife conservation, accurate sex identification is critical for American mammals with indistinct sexual dimorphism, yet relevant molecular studies are limited. In this study, the Y-chromosomal *SRY* gene, a core male sex-determining factor in mammals, was investigated. Primers were designed for its conserved regions, and PCR amplification, sequencing, and bioinformatic analysis were conducted on seven Neotropical mammals (e.g., two-toed sloth, jaguar). Their *SRY* nucleotide sequence similarity reached 83.2–95.0%. Over 60 American mammals from Carnivora, Xenarthra, Primates, Artiodactyla and Rodentia were further analyzed via GenBank data: a phylogenetic tree was built, primer binding sites were predicted, and the target fragment was found to have purifying selection (dN/dS = 0.352) and span three HMG-box motifs. This study provides a potential cross-species sex identification method for American mammals, offering molecular support for precise zoo management and wild conservation, with important practical and scientific value.

## Simple Summary

Accurately determining the sex of animals is a fundamental requirement for effective zoo management and wildlife conservation. However, many American mammals lack obvious physical differences between males and females, making visual identification challenging. This study addresses this problem by focusing on the *SRY* gene, a specific piece of genetic information found only in males that triggers male development. By designing specialized molecular tools to detect this gene, researchers analyzed various animals, including sloths and jaguars, and discovered that the gene’s structure was remarkably similar across these diverse species. The results confirmed that these genetic tools were highly effective for identifying the sex of seven Neotropical mammals and predicted sex identification primers for over 60 additional species in silico, including primates, xenarthrans, carnivores, artiodactyls and rodents. This research provides a reliable, non-invasive method for scientists to monitor animal populations accurately. By ensuring appropriate breeding pairs and well-balanced social groups, these findings offer essential support for protecting endangered wildlife and enhancing animal welfare in captivity, contributing significantly to the preservation of global biodiversity.

## 1. Introduction

Rapid and accurate DNA-based sex determination techniques serve as a cornerstone for sustainable captive wildlife husbandry and population management, playing a pivotal role in designing artificial breeding programs and advancing conservation research for endangered wildlife. Most animal species exhibit sexual dimorphism (SD), which refers to morphological differences between females and males, including variations in body size, coloration, and reproductive organs [1]. Although traditional sex determination heavily relies on these visual morphological traits, such methods exhibit significant limitations when applied to wildlife with indistinct physical SD, neonates, or when studies are restricted to non-invasively collected samples, such as hair or feces. With the development of molecular diagnostics technology, detection methods leveraging sex-specific genes have become a cornerstone in various medical and forensic applications. In most mammalian species, the *sex-determining region Y* (*SRY*) gene, located on the short arm of the Y chromosome, acts as the master testis-determining factor (TDF) that initiates male sex determination in therian mammals (i.e., placentals and marsupials) [2]. By contrast, monotremes adopt a different sex determination system. Owing to its high evolutionary conservation across diverse mammalian taxa [3], the development of polymerase chain reaction (PCR) assays targeting the *SRY* gene has emerged as a highly rapid, sensitive, and reliable molecular approach for mammalian sex identification [4,5,6,7].

In mammals, excluding monotremes and some rodents, females typically carry two X chromosomes, whereas males possess one X and one Y chromosome, with the Y chromosome being responsible for male sex determination. Since the identification of the *SRY* gene as the primary determinant of male sex in vertebrates in 1990, research has significantly advanced the understanding of the molecular mechanisms underlying sex determination and sexual development [8]. The SRY protein belongs to the high mobility group (HMG) protein family and has DNA-binding activity. It facilitates testis formation by stabilizing the SOX9 protein, thereby promoting Sertoli cell differentiation and subsequent testis cord development, which orchestrates the transformation of undifferentiated embryonic gonads into functional testes. Mutations or abnormal expression of the *SRY* gene can cause disorders of sex development (DSD), such as testicular hypoplasia or sex reversal [9,10]. Notably, although the core functional region of the *SRY* gene is relatively conserved, its flanking sequences exhibit pronounced evolutionary variations across different mammalian orders.

To facilitate sex identification in species lacking both distinct SD and published *SRY* gene reference sequences, this study aims to establish a PCR-based cross-species sex identification method. By utilizing conserved *SRY* gene fragments, we successfully validated this approach across seven Neotropical mammals, including the two-toed sloth (*Choloepus didactylus*), giant anteater (*Myrmecophaga tridactyla*), South American coati (*Nasua nasua*), maned wolf (*Chrysocyon brachyurus*), jaguar (*Panthera onca*), black-bearded saki (*Chiropotes satanas*), and pygmy marmoset (*Cebuella pygmaea*). This method offers a reliable and practical molecular tool for captive wildlife husbandry management and field conservation. Comparative analysis of the *SRY* nucleotide and protein sequences from these seven species unveiled their sequence features, thereby accumulating valuable data for studies on the molecular evolution and population genetics of American fauna.

## 2. Materials and Methods

### 2.1. Behavioral Training, Sample Collection and DNA Extraction

The sample collection process employed a progressive desensitization behavioral training approach. Positive reinforcement, comprising food rewards paired with clicker training, was integrated into routine grooming sessions over three months to facilitate stress-free hair plucking. Animal behavior was under continuous surveillance for any indicators of distress. Hair was collected during routine grooming sessions with the target animals, obtaining over 20 hairs in small quantities over multiple sessions. No significant stress responses or behavioral abnormalities were observed in the sloths during collection. Following collection, the hair samples were immediately transferred and stored at −80 °C. Hair samples were obtained from two sex-known two-toed sloths housed at Beijing Zoo. Liver samples of giant anteaters, jaguars, maned wolves, South American coati, black-bearded saki, and pygmy marmosets were obtained from historical animal mortality specimens preserved at Beijing Zoo, and these samples had been stored at −80 °C for over four years. Detailed information on all samples is listed in Table S1. Genomic DNA was extracted from hair and liver samples using the Tissue/Cell Genomic DNA Extraction Kit (Aidlab Co., Ltd., Beijing, China; Catalog: ND07) for extraction. DNA concentration was quantified using the Qubit^®^ dsDNA HS Assay Kit (Thermo Fisher Scientific, Waltham, MA, USA; Catalog: No. Q32854), and DNA integrity was assessed via agarose gel electrophoresis.

### 2.2. PCR and Sequencing

*SRY* universal primers were selected based on the classic primer pairs RG4 and RG7, which were designed targeting the conserved HMG-box domains of human and mouse [3,9,10]. The *SRY* nucleotide sequence of jaguar (GenBank accession No. DQ095180.1) and the *ACTBL2* nucleotide sequence of two-toed sloth (GenBank accession No. XM_037824592.1) were retrieved from the NCBI GenBank database (https://www.ncbi.nlm.nih.gov). Primer sequences and corresponding annealing temperatures are listed in Table 1. PCR amplifications were performed in a final volume of 25 μL, containing 10 ng of genomic DNA, 1 μL each of forward and reverse primer, and 12.5 μL of 2× Precise PCR Mix II (Zhongke Xinsheng (Beijing) Biotechnology Co., Ltd., Beijing, China; Cat. No. XS22001), supplemented with DEPC-treated water. The PCR amplifier (Thermo Fisher Scientific, Waltham, MA, USA; ABI 9700) was programmed as follows: 94 °C for 5 min; denaturation at 94 °C for 30 s; annealing at 56–58 °C for 30 s; extension at 72 °C for 30 s, repeated for 40 cycles; final extension at 72 °C for 10 min, followed by storage at 4 °C. We used 2% agarose gel electrophoresis to evaluate the efficacy of DNA extraction and amplification. Negative controls (Neg) were included in each PCR run using nuclease-free water. The gel was run to separate bands by molecular weight. The resulting PCR products were dispatched to Sangon Biotech Co., Ltd. (Shanghai, China) for subsequent purification and sequencing. Amplicons were purified using a gel extraction kit (BBI Life Sciences, Shanghai, China; Cat. No. B619602), followed by unidirectional sequencing on an ABI 3730XL DNA Analyzer (Thermo Fisher Scientific, Waltham, MA, USA) utilizing the forward primer.

### 2.3. Data Analysis

All phylogenetic analyses were conducted using MEGA7 [11]. Multiple sequence alignments were performed utilizing the MUSCLE algorithm. The best-fitting substitution model for each dataset was selected independently using the Find Best Model function, based on the Bayesian Information Criterion (BIC), such as the Kimura 2-parameter (K2) and Jones–Taylor–Thornton (JTT) models. Maximum likelihood (ML) trees were inferred for all datasets with 1000 bootstrap replicates. Initial trees for the heuristic search were obtained automatically by applying neighbor-joining (NJ) and BioNJ algorithms to a matrix of pairwise distances estimated using the respective default models, followed by selection of the topology with superior log-likelihood values. All positions containing gaps and missing data were eliminated using the complete deletion option. Branch support values ≥ 70% were considered well-supported. To visually illustrate sequence similarities and differences, multiple alignment results were submitted to the N.ESPript 0.9 web portal (https://nespript.ibcp.fr/ESPript/cgi-bin/NESPript.cgi, accessed on 20 May 2026) to generate a visualization of nucleotide sequence alignment diagrams. Homology analysis of *SRY* gene fragments from seven Neotropical mammals, human (GenBank Accession No. NM_003140.3), mouse (GenBank Accession No. NM_011564.1) and opossum (GenBank Accession No. XP_056665898.1) was performed using DNAStar v.7.1.0 software, confirming the evolutionary conservation of the *SRY* gene across different species, which was further confirmed by conducting a Basic Local Alignment Search Tool (BLAST, https://blast.ncbi.nlm.nih.gov, accessed on 4 January 2026) analysis on the *SRY* gene fragments from seven Neotropical mammals utilizing the NCBI GenBank database. We downloaded sequences and related information for high-similarity Carnivora, Xenarthra and Primates American animals (Table S2) and performed an analysis of the motif on the SRY protein sequences using the MEME online tool (https://meme-suite.org/meme, accessed on 21 May 2026) [12]. Conserved domain analysis was conducted using the CDD database (https://www.ncbi.nlm.nih.gov/Structure/bwrpsb/bwrpsb.cgi, accessed on 21 May 2026) [13], and the results were visualized using TBtools v2.476 [14]. Selection pressure analysis of *SRY* genes from 67 American mammals was performed using the nested models of the branch model (BM) and branch-site model (BSM) in EasyCodeml v1.41 [15]. The software estimates selective pressure by calculating the nonsynonymous-to-synonymous substitution rate ratio (ω = dN/dS). Genes evolving under neutral, purifying, and positive selection were characterized by ω = 1, 0 < ω < 1, and ω > 1, respectively. The amino acid sequences of *SRY* HMG-box domains from seven mammalian species (jaguar, bison, woodchuck, mouse, marmoset, sloth and opossum) were retrieved from the NCBI protein database. Three-dimensional homology modeling was conducted via the SWISS-MODEL web server (https://swissmodel.expasy.org) in automated mode [16]. For each target sequence, the SWISS-MODEL Template Library (SMTL) was searched using BLAST and HHblits to identify suitable template structures. Three-dimensional models were generated using the ProMod3 modeling engine, which builds models by transferring conserved coordinates from the templates and modeling insertions/deletions via loop modeling and side-chain reconstruction. For species with high sequence identity to the template, the automated mode was considered sufficient without manual intervention. Templates, global model quality estimation (GMQE), Ramachandran plot analysis, and the predicted local distance difference test (pLDDT) are shown in Table S3. Structural superimpositions between the species models and the reference human structure (PDB ID: 1J46) and root mean square deviation (RMSD) values were calculated using the PyMOL™ Molecular Graphics System 3.1.0 (https://pymol.org/).

## 3. Results

### 3.1. Amplification and Sequence Alignment of the SRY Gene in Seven American Mammals

**Figure 1:**
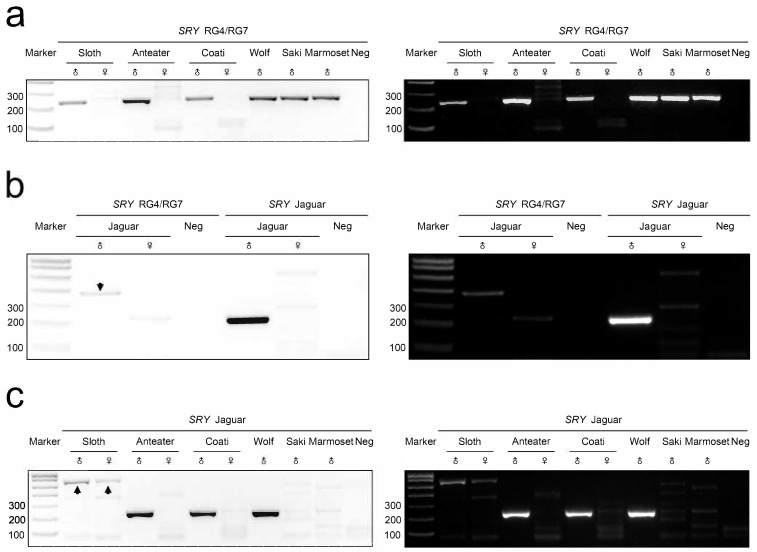
PCR amplification results of the *SRY* gene fragment in seven Neotropical mammals. Non-specific bands (observed in the male jaguar lane and the two-toed sloth lane at a higher molecular weight) are indicated by arrows. ♂: male, ♀: female. (**a**) *SRY* gene amplification using the classic *SRY* universal primers RG4/RG7 in six Neotropical mammals. (**b**) *SRY* gene amplification using the *SRY* RG4/RG7 primers and the newly designed *SRY* Jaguar primers in the male and female jaguar. (**c**) *SRY* gene amplification using the *SRY* Jaguar primers in six Neotropical mammals.

By employing primers *SRY* RG4/RG7, we successfully amplified the *SRY* gene fragments from the two-toed sloth, giant anteater, South American coati, maned wolf, black-bearded saki, and pygmy marmoset. Agarose gel electrophoresis resolved single, distinct bands slightly migrating above 200 bp exclusively in the males of these respective species (Figure 1a). Conversely, no detectable amplicons were observed in the female counterparts or the negative control. The amplification of the jaguar *SRY* gene fragment using primers *SRY* RG4/RG7 yielded no distinct positive bands. This amplification failure was provisionally ascribed to primer–template mismatches at the 3′ end or internal primer-binding regions, a common issue when human-derived primers are applied to distantly related mammalian orders. The jaguar belongs to Carnivora, whereas the RG4/RG7 primers were originally designed based on *SRY* sequences from human and mouse [3]. Platyrrhine primates preceded terrestrial carnivorans in South America by approximately 33 to 40 million years [17,18], which logically accumulated nucleotide substitutions within the primer binding regions. To circumvent this limitation, alternative primers (*SRY* Jaguar) were designed using the jaguar *SRY* reference sequence (GenBank accession DQ095180.1). Subsequent assays demonstrated that the *SRY* Jaguar primers successfully yielded the expected sex-specific positive bands in the male jaguar, with a total absence of amplification in both the female jaguar and the negative control (Figure 1b). To further validate the specificity of the newly designed *SRY* Jaguar primers across a broader taxonomic range, additional mammalian DNA samples were screened. As shown in Figure 1c, positive bands were observed in the male giant anteater, South American coati and maned wolf. In contrast, two-toed sloth samples exhibited a prominent high-molecular-weight non-specific band, while no amplicons of the expected size were observed. This suggests that the *SRY* Jaguar primer binding sites are not conserved in sloth, possibly due to lineage-specific indels or substitutions within the primer annealing domains. Overall, the *SRY* Jaguar primers successfully amplified the target fragment in four of the seven tested species (jaguar, giant anteater, South American coati, maned wolf), demonstrating broad but not universal utility across Xenarthran and Carnivoran taxa. To confirm the successful DNA extraction from the above species, *ACTBL2* was employed as an internal control. The results are presented in Figure S1. The male and female two-toed sloth, giant anteater, black-bearded saki, and pygmy marmoset exhibited positive bands, while weak positive bands were observed in the South American coati, maned wolf, and jaguar, indicating limited primer universality across the studied species. This is not unexpected given that *ACTBL2* is a nuclear-encoded gene; while generally conserved, primer binding sites may still accumulate lineage-specific substitutions over evolutionary timescales spanning millions of years [19].

**Figure 2:**
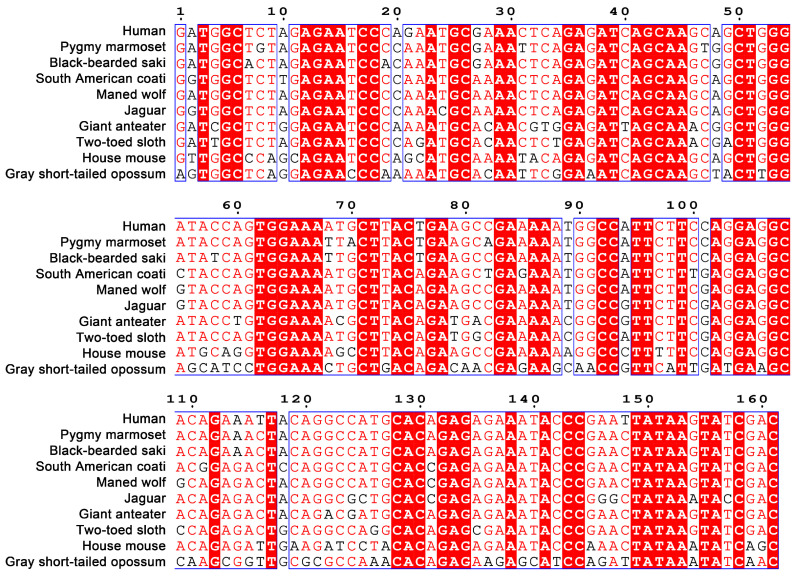
Comparison of *SRY* gene fragments among seven Neotropical mammals, human, mouse and opossum. Identical bases are highlighted in white on a red background; majority-identical bases are highlighted in red on a white background; differing bases are highlighted in black against a white background. The bases within the blue rectangle form a highly similar continuous sequence.

Sequencing of PCR amplification products from the above animal species was performed and compared against the *SRY* genes of humans (NM_003140.3) and mice (NM_011564.1) (Figure 2). Among the aligned bases, 61% (98/161) of the bases were invariant, 31% (50/161) displayed majority identity, and 8% (13/161) were divergent. Homology analysis (Table 2) revealed similarity ranging from 83.2% to 95.0% among the seven Neotropical mammals. These values indicate moderate sequence conservation, consistent with the well-established functional constraints acting on the *SRY* HMG box; however, such conservation should not be overinterpreted due to the length of the targeted fragment (161 bp). The black-bearded saki and the pygmy marmoset exhibited the least divergence, whereas the greatest divergence was found between the giant anteater and the pygmy marmoset. The gray short-tailed opossum (*Monodelphis domestica*, GenBank Accession No. XP_056665898.1) showed relatively lower similarity, ranging from 61.5% to 66.5%, and the greatest genetic distance compared to other species.

**Figure 3:**
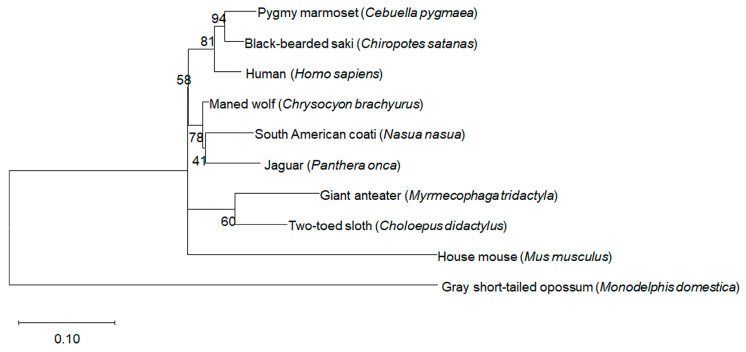
An ML phylogenetic tree of *SRY* gene fragments from seven Neotropical mammals, human, mouse and opossum with MEGA7. Nucleotide sequences were aligned using MUSCLE. The best-fitting model was [K2+I], selected by the BIC. The tree with the highest log likelihood (−806.26) is shown. The tree is drawn to scale, with branch lengths measured in the number of substitutions per site. All positions containing gaps and missing data were eliminated. There were a total of 161 positions in the final dataset. The sequence of gray short-tailed opossum (GenBank Accession No. XM_056809920.1) was used as the outgroup.

Using the opossum as the outgroup, a phylogenetic tree was constructed via the ML method (Figure 3). The pygmy marmoset, black-bearded saki, and human clustered into one clade; the jaguar, South American coati, and maned wolf formed a second clade; and the giant anteater and two-toed sloth constituted a third clade. This clustering pattern is similar to the taxonomic classification of the orders Primates, Carnivora, and Xenarthra, respectively. Notably, some branches exhibited low bootstrap support values (typically <70%). The short target fragment length (161 bp) and high sequence similarity among the studied taxa substantially limited the phylogenetic resolution of the tree. Therefore, the inferred branching topology should be interpreted with caution, and no robust phylogenetic claims beyond the ordinal level are warranted.

### 3.2. Sequence Alignment of the SRY Gene and Its Corresponding Protein Among American Mammals

BLAST alignment of *SRY* gene fragments from the seven Neotropical mammals identified closely matching representative species across multiple eutherian orders, spanning Xenarthra (n = 4), Carnivora (n = 35), and Primates (n = 19). The nucleotide sequences of the two-toed sloth, giant anteater, jaguar, and pygmy marmoset exhibited a striking resemblance to their respective sequencing outcomes, with percentage identities ranging from 97.67% to 98.84% and query coverage rates between 86% and 93% (Tables S4–S7). The high similarity between the black-bearded saki and the pygmy marmoset is entirely consistent with their close phylogenetic affinity within the New World monkeys (Platyrrhini). The *SRY* nucleotide sequences of the South American coati, maned wolf, and black-bearded saki were absent from the NCBI database GenBank. The results indicated that the sequences showing the highest percent identity to the South American coati *SRY* gene fragment sequence were leopard seal (*Hydrurga leptonyx*, GenBank Accession No. AY424655.1), Weddell seal (*Leptonychotes weddellii*, GenBank Accession No. KY608931.1) and southern elephant seal (*Mirounga leonina*, GenBank Accession No. AY424657.1), with a percent identity of 93.22% and query coverage of 92% (Table S8). This may be due to the short length of the fragments and the lack of genetic data from the Procyonidae family. The sequence with the highest percent identity to the maned wolf’s *SRY* gene fragment sequence was that of the Mexican gray wolf (*Canis lupus baileyi*, GenBank Accession No. XM_072819280.1), showing 97.63% identity and 87% query coverage (Table S9). The sequence with the highest percent identity to the black-bearded saki *SRY* gene fragment sequence was found in 16 sequences, including those from the families Cebidae, Aotidae, and Callitrichidae, with a percent identity of 96.32% and a query coverage of 84% (Table S10).

**Figure 4:**
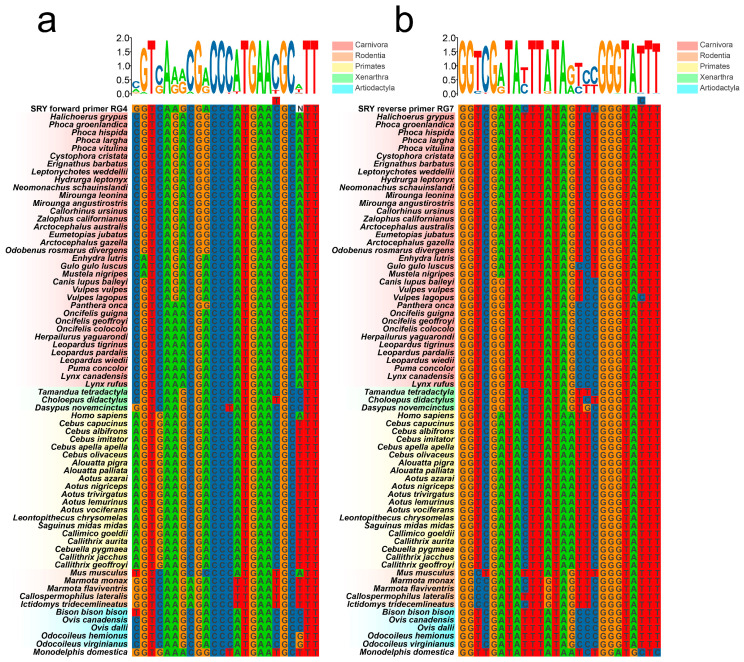
Predicted *SRY* primers for 67 American mammals. (**a**) represents forward primers; (**b**) represents reverse primers.

The *SRY* nucleotide sequence GenBank Accession No. EU797119.1 from the giant anteater was too short to predict primers. Based on primers *SRY* RG4/RG7 and *SRY* Jaguar, primers for the remaining 67 American mammals, human, and mouse were predicted. The predictive modeling (Figure 4) revealed that, while primer configurations exhibit family-level conservation among marine carnivores and non-human primates, pronounced sequence heterogeneity manifests within the xenarthrans. In a comparative study of *SRY* gene primers, the forward primer *SRY* RG4 exhibited base changes at positions 1 (G→C), 7 (G→A), and 10 (A→G), while the reverse primer *SRY* RG7 had alterations at positions 6 (A→G) and 9 (C→T), and two at positions 16 and 17 (both T→C). These positional differences might reduce primer adaptability. Analysis combining the predicted primers for the two-toed sloth and pygmy marmoset data suggested that the number of differing bases, especially at positions 7 and 10 in the forward primer and positions 9 and 16 in the reverse primer, might significantly influence primer adaptability.

**Figure 5:**
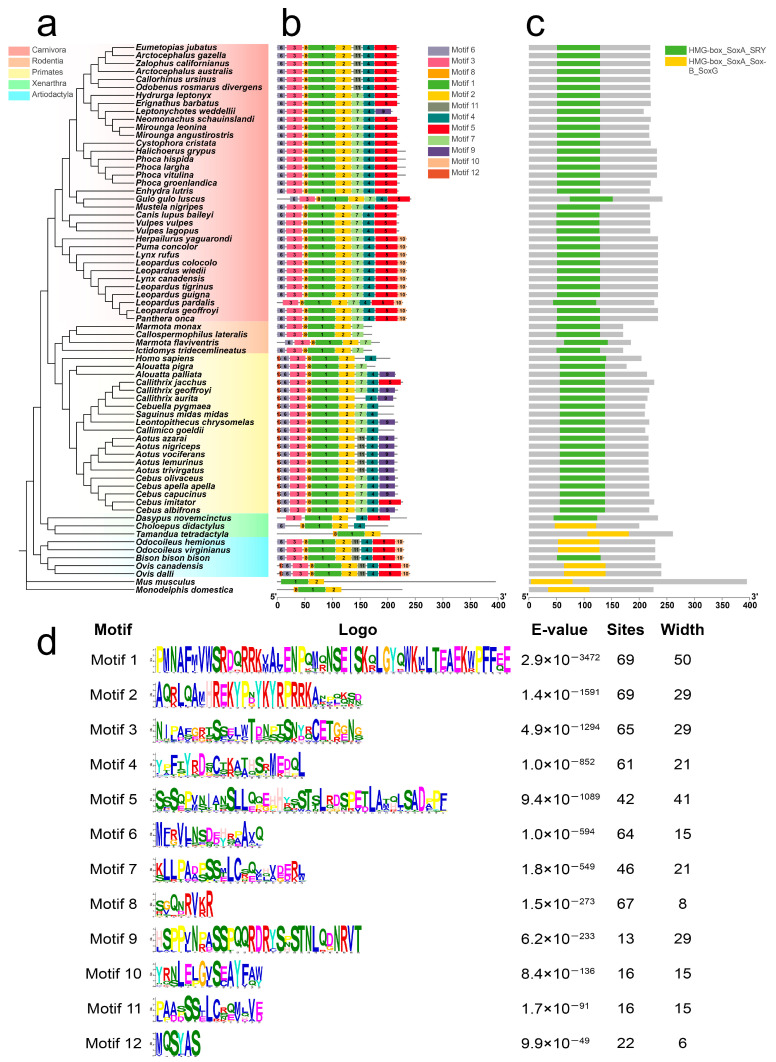
Motif analysis and conservation domain analysis of *SRY* protein sequences from 67 American mammals. (**a**) An ML phylogenetic tree of *SRY* based on amino acid sequences from American mammals with MEGA7. Amino acid sequences were aligned using MUSCLE. The best-fitting model was [JTT+G], selected by the BIC. The tree with the highest log likelihood (−1617.16) is shown. All positions containing gaps and missing data were eliminated. There was a total of 97 positions in the final dataset. The sequence of gray short-tailed opossum (GenBank Accession No. XP_056665898.1) was used as the outgroup. (**b**) Motif localization; (**c**) conservation domain localization; (**d**) motif conservation, reliability, sites, and width. In the annotation, letters of varying height and color represent amino acid types and the predicted reliability of each motif.

An ML phylogenetic tree was constructed based on 67 sequences as well as the *SRY* amino acid sequences from humans and mice. The reconstruction was rooted using the opossum (GenBank Accession No. XP_056665898.1) as the outgroup (Figure 5a). The clustering topology was similar to traditional taxonomic classifications. Motif analysis revealed conserved characteristics across the *SRY* amino acid sequences, with the highest conservation observed within the HMG domain, a functionally critical region that governs testicular differentiation initiation. The positional information, conserved sites, conservation levels, and reliability statistics of each motif are shown in Figure 5d. All sequences contained Motif 1 and Motif 2, which were the most reliable (Motif 1 E-value: 2.9 × 10^−3472^, Motif 2 E-value: 1.4 × 10^−1591^). Motif 8 was detected in 97.1% (67/69) of sequences with high confidence (E-value: 1.5 × 10^−273^). Remarkably, even highly divergent taxa, such as members of the families Phocidae and Didelphidae, share a conserved architecture comprising Motif 8, Motif 1, and Motif 2 (Figure 5b), which map precisely into the structural HMG-box domain (Figure 5c), indicating convergent evolution of motifs and relative conservation among the studied mammalian species. The *SRY* RG4/RG7 and *SRY* Jaguar primers were designed based on this conserved HMG-box domain. Given that *SRY* is a strictly Y-linked, male-specific marker, validated genomic amplification serves as a dependable diagnostic for molecular sexing. However, this fragment exhibits limited cross-species applicability, as primer binding efficiency varies among taxa and successful amplification is not guaranteed for all species (e.g., weak amplification in the male jaguar with primers *SRY* RG4/RG7 and non-specific product in the sloths with primers *SRY* Jaguar). Therefore, any application of this *SRY* fragment for sex determination in uncharacterized species requires empirical validation of primer specificity for each target taxon.

### 3.3. Selection Pressure Analysis and Structural Modeling of the SRY Gene in American Mammals

The BM analysis (Table 3) showed that dN/dS = 0.352 for the *SRY* gene in American animals under Model 0, indicating that the *SRY* gene has evolved under purifying selection in these species. Two-Ratio Model 2 showed no significant difference in selective pressure acting on branches corresponding to Carnivores, Artiodactyls, Xenarthrans, non-mouse rodents, and non-human primates relative to the overall phylogenetic background (*p* > 0.05). To further verify the presence of positive selection acting on the *SRY* gene, the BSM was applied (Table 4). For the likelihood ratio test (LRT), *p* > 0.05 for all American animal *SRY* genes, indicating no significant difference between Model A and the null Model A. In the Artiodactyla branch, potential positive selection sites were detected at position 36 (glutamic acid, E; posterior probability [PP] = 0.525) and position 51 (glutamic acid, E; PP = 0.513). In the non-mouse Rodentia branch, a potential positive selection site was identified at position 4 (methionine, M; PP = 0.589). All PP values were below 0.95, indicating no statistically significant evidence for positive selection. Owing to the truncated alignment configuration (289 positions), these selective pressure parameters must be evaluated with discretion, as short sequences often provide limited phylogenetic signal and lack the statistical power to resolve episodic adaptive evolution.

**Figure 6:**
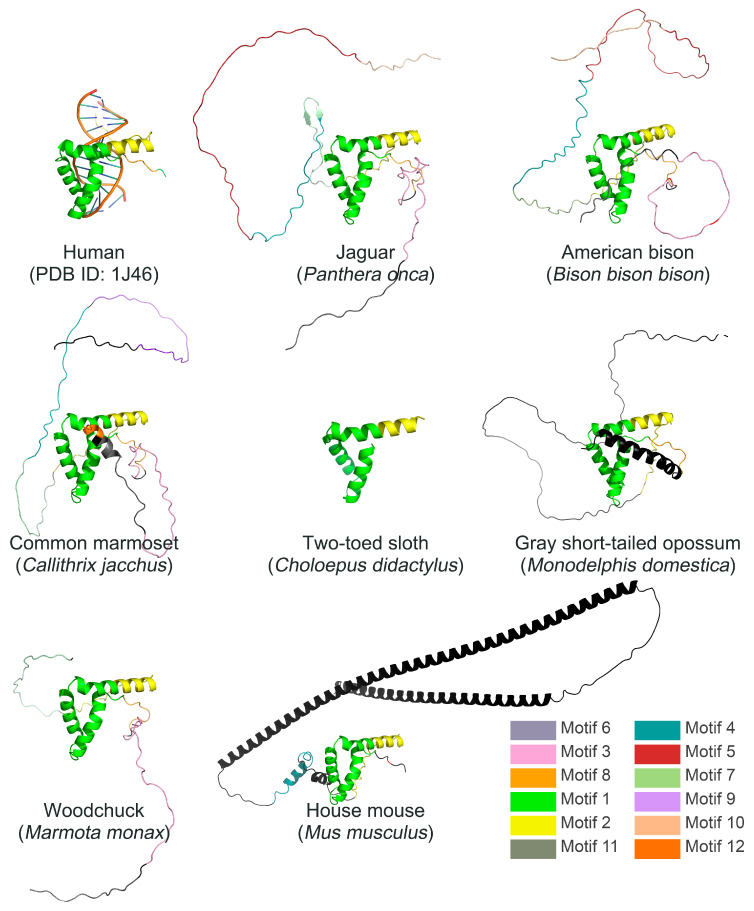
Structural comparison of *SRY* HMG-box domains across multiple mammalian species. The *SRY* HMG-box domain structures were predicted with the SWISS-MODEL web server from seven species: jaguar (GenBank Accession No. ABA64501.1), American bison (GenBank Accession No. XP_010858437.1), common marmoset (GenBank Accession No. ACL51659.1), two-toed sloth (GenBank Accession No. XP_037680520.1), gray short-tailed opossum (GenBank Accession No. XP_056665898.1), woodchuck (GenBank Accession No. XP_046276129.1) and house mouse (GenBank Accession No. NP_035694.1). All structures were aligned to the human template structure (PDB ID: 1J46) using the PyMOL™ Molecular Graphics System 3.1.0.

Three-dimensional homology models of the *SRY* HMG-box domain were successfully resolved for eight representative species. The majority of the generated structural coordinate files exhibited high overall fold fidelity, with GMQE scores spanning 0.58 to 0.68. An exception was observed for the two-toed sloth model (GMQE = 0.24), which exhibited significantly depressed template coverage (28%) due to an absence of high-identity target structures in the repository (Table S3). Although the Ramachandran plot statistics were slightly suboptimal compared with experimental structures, the consistently low RMSD values (0.704–0.963 Å) relative to the high-resolution 1J46 template support the overall fold accuracy of all models despite some local geometric deviations. To focus on the core regions, the average pLDDT scores for the HMG-box domains were calculated. The majority of species showed average pLDDT > 0.95, with more than 91.18% of residues falling into the very-high-confidence range (pLDDT > 0.9), reflecting exceptional stereochemical reliability within the predicted local structures. The lower confidence observed for the sloth model likely stems from its low template coverage (28%) rather than inherent structural divergence. The predicted tertiary structure of the *SRY* HMG-box domain is shown in Figure 6. As expected, the fold consists of three α-helices arranged in an ‘L’-shaped conformation, a characteristic of the HMG-box family of DNA-binding proteins. The HMG-box domain (approximately residues 60–140 across species) consists primarily of Motif 1 and Motif 2. The putative positive selection sites 4M, 36E and 51E are all located outside the DNA-binding plane, suggesting that mutations at these sites have minimal impact on DNA-binding ability. Nevertheless, further research is required to determine whether localized structural variations—such as the β-sheet motif resolved in the jaguar model, or the divergent α-helical extensions in the marmoset, opossum, and mouse architectures—induce steric hindrance or modulate DNA-bending affinity.

## 4. Discussion

The long period of geographical isolation in South America provides an excellent natural laboratory for studying how mammals evolved and diversified [20]. Fossil evidence indicates that colonization of exogenous taxa has increased overall species richness while decreasing native species diversity, reflecting competition-driven diversity replacement [21]. Although phylogenetic studies have clearly described the evolutionary history of major biogeographic units in this region [22], an important question remains unanswered: whether these historical barriers and evolutionary events influenced the molecular evolution of core sex-determining proteins in mammals. As shown in Figure 5 and Figure 6, the HMG-box domain is highly conserved across the 67 studied American mammal species, as well as in humans and mice. Most of these sequences share a common structural framework consisting of three main motifs, namely Motif 8, Motif 1, and Motif 2. Notably, the nine-banded armadillo (*Dasypus novemcinctus*) and the mouse lack Motif 8. Given that the gene database contains only one amino acid sequence for the nine-banded armadillo, more biological samples are needed to determine whether this absence is a real evolutionary change or just an error from incomplete sequence data. In mice, the N-terminal domain of the *SRY* protein is restricted to two amino acids; concurrently, the C-terminal domain has undergone compensatory evolution, in which the C-terminal polyglutamine (polyQ) domain functionally compensates for the N-terminal deficiency via two mechanisms: maintenance of protein stability and transcriptional activation [23]. Motif 12 and Motif 6 at the N-terminus, as well as Motif 10, Motif 5 and Motif 9 at the C-terminus, are easily affected by detection methods and the completeness of the input data; therefore, they are not discussed further. Interestingly, the Otariidae and Phocidae families within Carnivora, the Aotidae family within Primates, and the Bovidae and Cervidae families within Artiodactyla all exhibit Motif 11 in place of Motif 7, despite differences in their geographic distributions and lifestyles. Further research on the specific functions of these motifs and motif convergence analysis could be conducted using techniques such as dCas9-controlled CRISPR/Cas3 [24].

In human clinical practice, many *SRY* mutations cause gonadal dysgenesis or DSD by affecting protein spatial conformation and DNA-binding ability. Similar cases are occasionally observed in veterinary practice [21,25,26]. Based on the high structural similarity found in the HMG-box domain in our study, we mapped known human mutation sites [27,28,29,30,31,32,33] onto our motif analysis. As shown in Figure 5, position 8 in motif 8 (human R62Q); positions 2 (M64T), 8 (W70L), 13 (R75N), 14 (R76P/S), 23 (M85T), 32 (L94R), 33 (G95R), and 36 (W98R) in motif 1; and positions 15 (Y127C) and 21 (R133W) in motif 2 are all highly conserved across the studied American mammals. Whether similar mutations cause the same gonadal abnormalities or DSD phenotypes in wild American mammals needs to be confirmed by future clinical and field case evidence. This speculation has its limitations. The gene fragments and amino acid sequences analyzed in this study are relatively short, which limits the power of the phylogenetic analysis and selection pressure calculations. Therefore, before applying this method for sex determination in other wild animals, validation using individuals of known sex is necessary. Furthermore, *SRY* expression is controlled by a complex regulatory network including *Gata4*, *zinc finger proteins*, *Zfpm2*, *Wt1*, *Nr5a1*, *Cbx2*, *Gadd45g*, and *Map3k4* and so on. Mutations in these genes down-regulated *SRY* mRNA and protein levels, leading to varying degrees of male-to-female sex reversal [8]. Recent studies have demonstrated that the *SDX* gene on the X chromosome cooperates with the *SRY* gene on the Y chromosome to ensure male sex determination, as evidenced by the sex reversal observed in male mice with *SDX* gene mutations [34]. Single identification of the *SRY* gene cannot distinguish sex reversal caused by sex chromosome abnormalities, dysregulation of positive regulatory factors, or mutations in X-linked genes. Therefore, molecular marker-based sex identification methods require further refinement.

In addition to genetic factors, the sex determination of captive wild animals is strongly influenced by sample types and preservation approaches. To ensure animal welfare, we collected hair samples from live animals through behavioral training, as well as frozen samples from animals that died naturally. Sample quality was verified via agarose electrophoresis (Figures S2–S5) and reference gene amplification from extracted DNA (Figure S1). Despite tailing in some positive bands, we successfully amplified approximately 200 bp fragments of both the *ACTBL2* and *SRY*. For the giant anteater, we compared liver samples stored for more than four years with fresh hair samples. The DNA bands on the gel were similar between the two sample types; however, the DNA yield from frozen liver was much higher than that from hair follicles, which agrees with previous reports [35]. To keep DNA stable during long-term storage at −80 °C or in liquid nitrogen, repeating freeze–thaw cycles must be avoided [36], and using a nucleic acid carrier during extraction improves stability [37]. Although sexing with feces and oral swabs was tested, the results were limited by fecal bacteria and low DNA content in oral swabs, respectively. If combined with advanced DNA extraction techniques [38,39], such as the AllPrep DNA/RNA Mini Kit and the QIAamp Fast DNA Stool Mini Kit, to improve detection sensitivity, it may be possible to achieve efficient testing of non-invasive samples, thereby providing strong support for the investigation of sex structure and conservation genetics research on wild American animal populations.

For endangered North American carnivores like the Pacific marten (*Martes caurina*), Tucker et al. developed footprint recognition technology, achieving over 90% accuracy in sex determination, offering a non-invasive and cost-effective method for monitoring sex-specific habitat requirements (e.g., female nursery habitats) [40]. Similarly, Merchan et al. employed machine learning to analyze acoustic signatures of Caribbean manatees, achieving 85–87% accuracy in sex classification, providing a new way to study population structure in aquatic mammals that are difficult to observe [41]. In biology conservation practice, precise sex identification is crucial for endangered species management, as significant differences often exist between sexes in habitat use, diet and reproductive requirements. Combined with genetic sex identification methods, these non-invasive field techniques successfully overcome the problems of unclear external physical differences between sexes, creating a solid scientific foundation for precise conservation strategies. It is noteworthy that animal breeding success rates are not only related to sex matching but are also regulated by multiple factors. Taking the two-toed sloth as an example, its chromosome number exhibits significant variation (2n = 53–67) [42]. Meanwhile, environmental conditions [43], growth and development status, nutritional levels, behavioral patterns, and social relationships [44] can all contribute to breeding failure in sloths, and comprehensive studies incorporating multi-dimensional factors are required in subsequent research.

## 5. Conclusions

This study successfully amplified the *SRY* gene fragments from seven Neotropical mammals, including the two-toed sloth, giant anteater, South American coati, maned wolf, jaguar, black-bearded saki, and pygmy marmoset, using the universal *SRY* RG4/RG7 and species-specific *SRY* Jaguar primer sets. Nucleotide sequence similarity > 80%, with dN/dS < 0.4, indicating high sequence conservation, subject to purifying selection, and suggesting potential utility for cross-species sex identification. Based on reference database sequences, we in silico predicted functional *SRY* primers for more than 60 additional American mammals across Carnivora, Xenarthra, Artiodactyla, Rodentia, and Primates. This work establishes a practical and dependable molecular marker-based sex determination framework for American mammals, offering valuable technical support for captive zoo management, artificial breeding programs, and wildlife conservation genetics.

## Figures and Tables

**Table 1:** Primer sequences.

Name	Primer	Product Length (bp)	Annealing Temperature (°C)
*SRY*	Forward RG4: GGTCAAGCGACCCATGAA(C/T)GCNTT Reverse RG7: GGTCGATACTTATAGTTCGGGTA(C/T)TT	216	56
*SRY* Jaguar	Forward: CGTCAAACGGCCCATGAACGCATT Reverse: GGTCGGTATTTATAGCCCGGGTATTT	216	58
*ACTBL2*	Forward: TCCTGACAGAGCGAGGCTAT Reverse: AGGGCATCGGAAACGTTCAT	200	56

**Table 2:** Homology analysis of seven Neotropical mammals, human, mouse and opossum.

Species	Human	Marmoset	Saki	Coati	Wolf	Jaguar	Anteater	Sloth	Mouse	Opossum
Human	***	93.2	94.4	89.4	93.2	88.2	85.1	86.3	81.4	64.6
Marmoset	7.2	***	95	88.2	91.3	86.3	83.2	85.7	77.6	61.5
Saki	5.8	5.1	***	88.8	92.5	87.6	84.5	87	77.6	63.4
Coati	11.5	12.9	12.1	***	94.4	90.7	83.9	86.3	77.6	65.2
Wolf	7.2	9.3	7.9	5.8	***	93.8	87	90.1	79.5	64.6
Jaguar	13	15.3	13.7	10	6.6	***	84.5	84.5	78.3	64
Anteater	16.8	19.1	17.5	18.3	14.4	17.7	***	88.2	77	65.2
Sloth	15.1	15.9	14.3	15.1	10.7	17.5	12.9	***	75.2	66.5
Mouse	21.7	26.8	26.8	26.8	24.2	26.1	27.7	30.3	***	63.4
Opossum	50.1	57	53	47.9	50.1	50.9	49.5	46.7	50.9	***

***: the dividing line between Percent Similarity and Percent Divergence. Percent Similarity is shown in the upper triangle. Percent Divergence is shown in the lower triangle.

**Table 3:** The selection pressure analysis of *SRY* genes in American mammals based on BM.

Model	Foreground Branch	ω0	ω1	LRT *p*-Value
Model 0		0.352		
Two ratio Model 2	Carnivora	0.359	0.077	0.106
	Artiodactyla	0.350	0.398	0.803
	Non-mouse Rodentia	0.351	0.368	0.941
	Non-human Primates	0.348	0.502	0.404
	Xenarthra	0.350	0.466	0.814

An ML phylogenetic tree of *SRY* from American mammals with MEGA7. Nucleotide sequences were aligned using MUSCLE (codon). The best-fitting model was [K2+G], selected by the BIC. The tree with the highest log likelihood (−2759.80) is shown. All positions containing gaps and missing data were eliminated. There were a total of 289 positions in the final dataset. The sequence of gray short-tailed opossum (GenBank Accession No. XM_056809920.1) was used as the outgroup. ω0 denotes the dN/dS ratio of the background branch, while ω1 denotes the dN/dS ratio of the foreground branch.

**Table 4:** The selection pressure analysis of *SRY* genes in American mammals based on BSM.

Foreground Branch	Model	Estimates of Parameters					LRT *p*-Value	Positive Sites (PP)
		Site Class	0	1	2a	2b		
Carnivora	Model A	f	0.641	0.359	0.000	0.000		
		ω0	0.071	1.000	0.071	1.000		
		ω1	0.071	1.000	1.000	1.000		
	Model A null	1					1.000	Not Allowed
Artiodactyla	Model A	f	0.553	0.294	0.100	0.053		36 E (0.525) 51 E (0.513)
		ω0	0.070	1.000	0.070	1.000		
		ω1	0.070	1.000	1.000	1.000		
	Model A null	1					1.000	Not Allowed
Non-mouse	Model A	f	0.641	0.359	0.000	0.000		4 M (0.589)
Rodentia		ω0	0.071	1.000	0.071	1.000		
		ω1	0.071	1.000	5.645	5.645		
	Model A null	1					1.000	Not Allowed
Non-human	Model A	f	0.641	0.359	0.000	0.000		
Primates		ω0	0.071	1.000	0.071	1.000		
		ω1	0.071	1.000	1.000	1.000		
	Model A null	1					0.998	Not Allowed
Xenarthra	Model A	f	0.641	0.359	0.000	0.000		
		ω0	0.071	1.000	0.071	1.000		
		ω1	0.071	1.000	1.000	1.000		
	Model A null	1					0.331	Not Allowed

An ML phylogenetic tree of *SRY* from American mammals with MEGA7. Nucleotide sequences were aligned using MUSCLE (codon). The best-fit nucleotide substitution model, K2+G, was selected by the BIC. The tree with the highest log likelihood (−2759.80) is shown. The sequence of gray short-tailed opossum (GenBank Accession No. XM_056809920.1) was used as the outgroup. There were four categories of sites: 0 denotes purifying selection, 1 denotes neutral selection, and 2a and 2b denote positive selection. ω0 represents the dN/dS ratio of the background branch, and ω1 represents the dN/dS ratio of the background branch.

## Data Availability

The datasets generated and analyzed during the current study are available from the corresponding author on reasonable request.

## Supplementary Materials

The following supporting information can be downloaded at: https://www.mdpi.com/article/10.3390/ani16131949/s1, Figure S1: PCR amplification results of the *ACTBL2* gene fragment in seven Neotropical mammals.; Figures S2–S5: electrophoresis gel graph for evaluating sample quality; Table S1: sample information and DNA content; Table S2: American animals’ mRNA and protein accessions of *SRY*; Table S3: homology modeling and validation; Table S4: the two-toed sloth *SRY* gene alignment in American mammals; Table S5: the giant anteater *SRY* gene alignment in American mammals; Table S6: the jaguar *SRY* gene alignment in American mammals; Table S7: the pygmy marmoset *SRY* gene alignment in American mammals; Table S8: the South American coati *SRY* gene alignment in American mammals; Table S9: the maned wolf *SRY* gene alignment in American mammals; Table S10: the black-bearded saki *SRY* gene alignment in American mammals.

## Abbreviations

The following abbreviations are used in this manuscript:

SD	Sexual dimorphism
*SRY*	Sex-determining region Y
TDF	Testis-determining factor
PCR	Polymerase chain reaction
HMG	High mobility group
DSD	Disorders of sex development
BLAST	Basic Local Alignment Search Tool
NCBI	National Center for Biotechnology Information
NJ	Neighbor-Joining
ME	Minimum Evolution
BM	Branch model
BSM	Branch-site model
dN/dS	The ratio of non-synonymous to synonymous substitutions
GMQE	Global model quality estimation
pLDDT	Predicted local distance difference test
RMSD	Root mean square deviation
LRT	Likelihood ratio test
PP	Posterior probability
