# Supplementary figures and images for: Cross-Species Sex Identification and Comparative Analysis of the SRY Gene in American Mammals
Source: Animals (Basel). 2026 Jun 23;16(13):1949. doi: 10.3390/ani16131949 (PMC13360611; doi:10.3390/ani16131949)

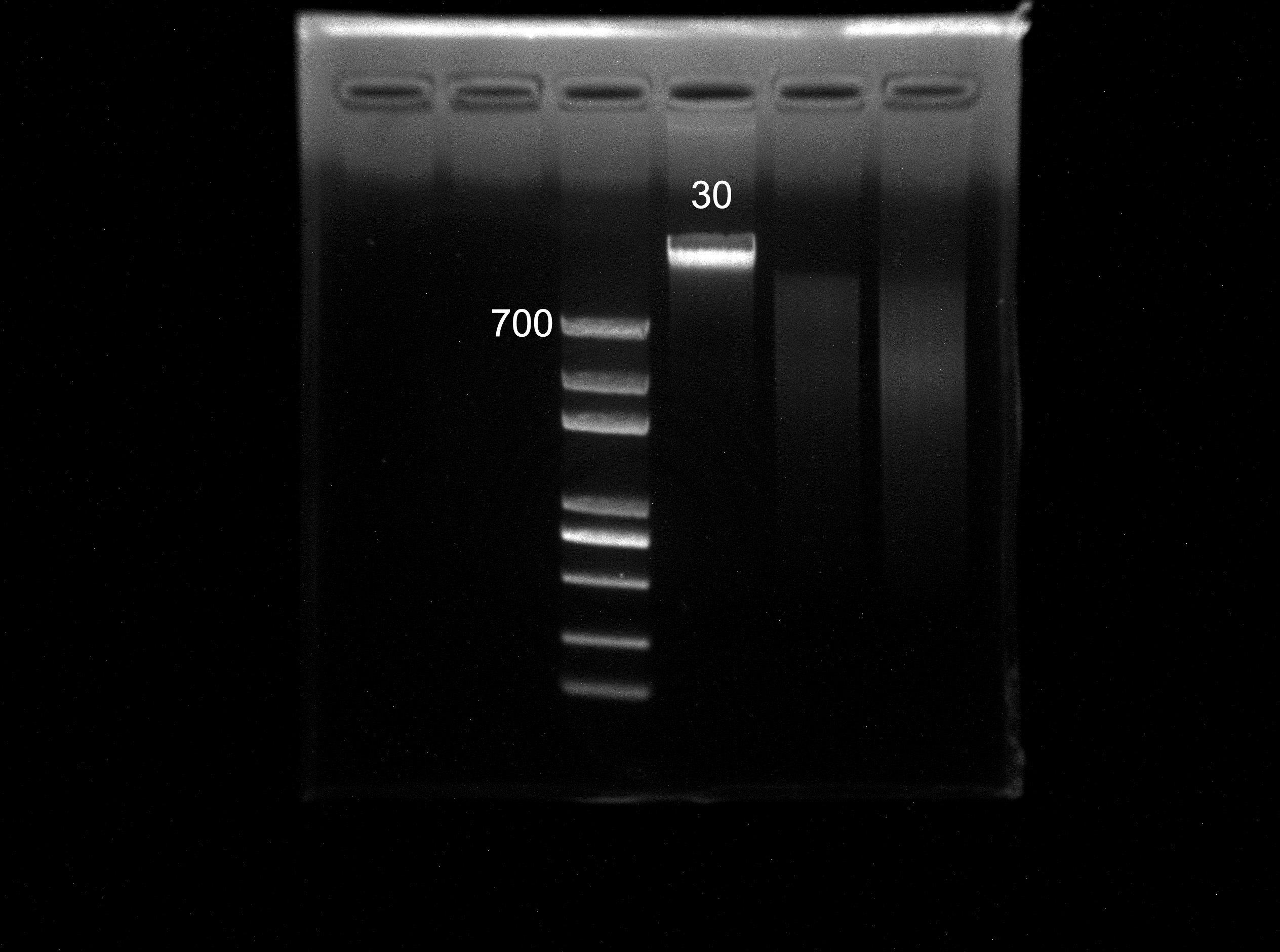

Supplement: Supplementary file 1 [file animals-16-01949-s001.zip › Figure S5.jpg]

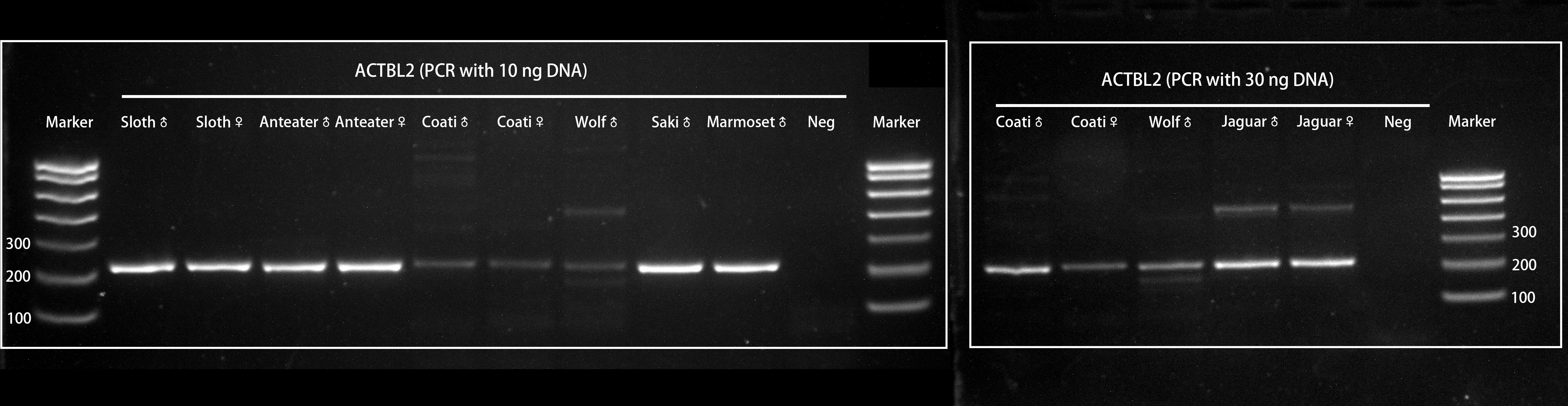

Supplement: Supplementary file 1 [file animals-16-01949-s001.zip › Figure S1.jpg]

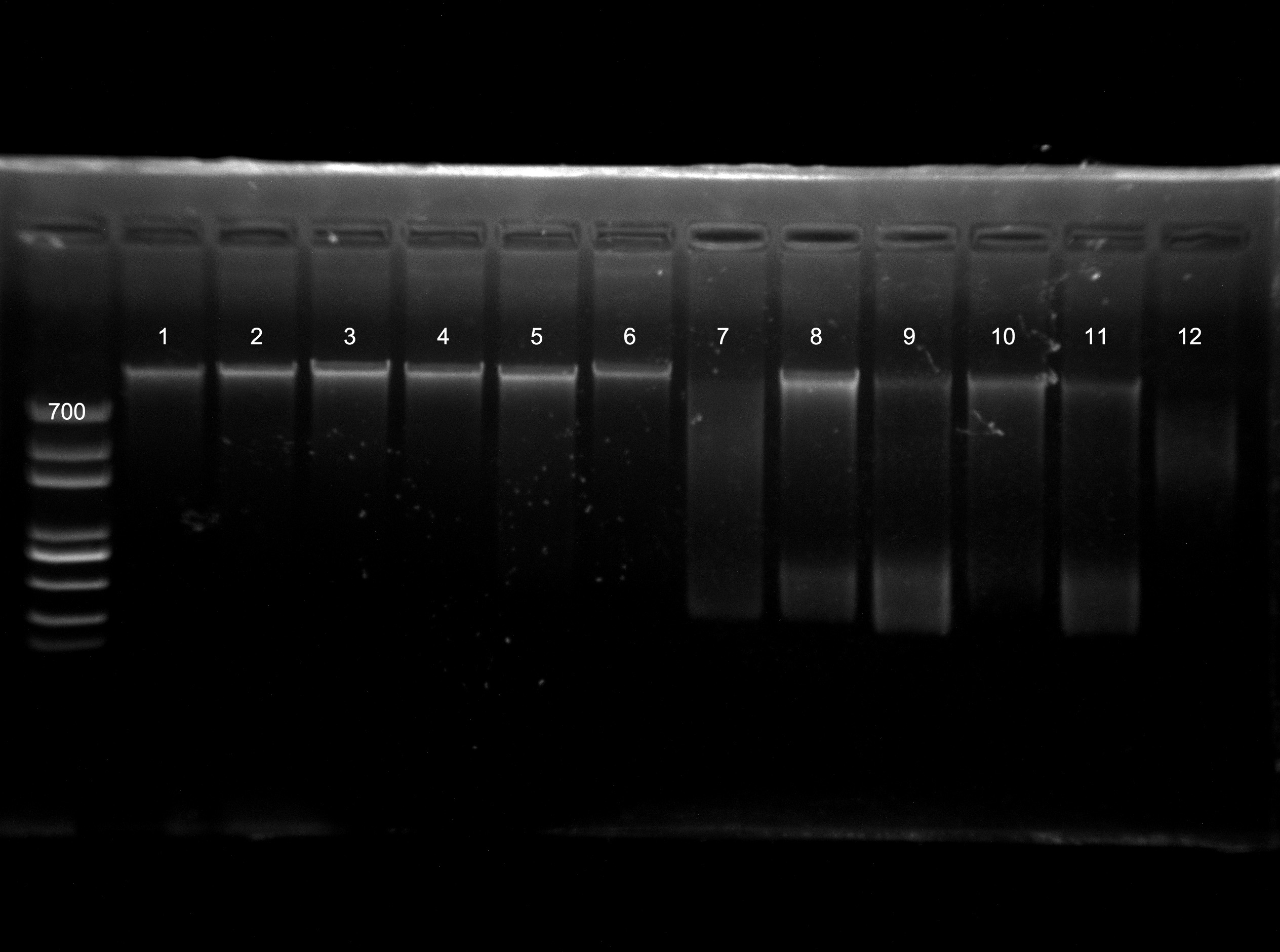

Supplement: Supplementary file 1 [file animals-16-01949-s001.zip › Figure S2.jpg]

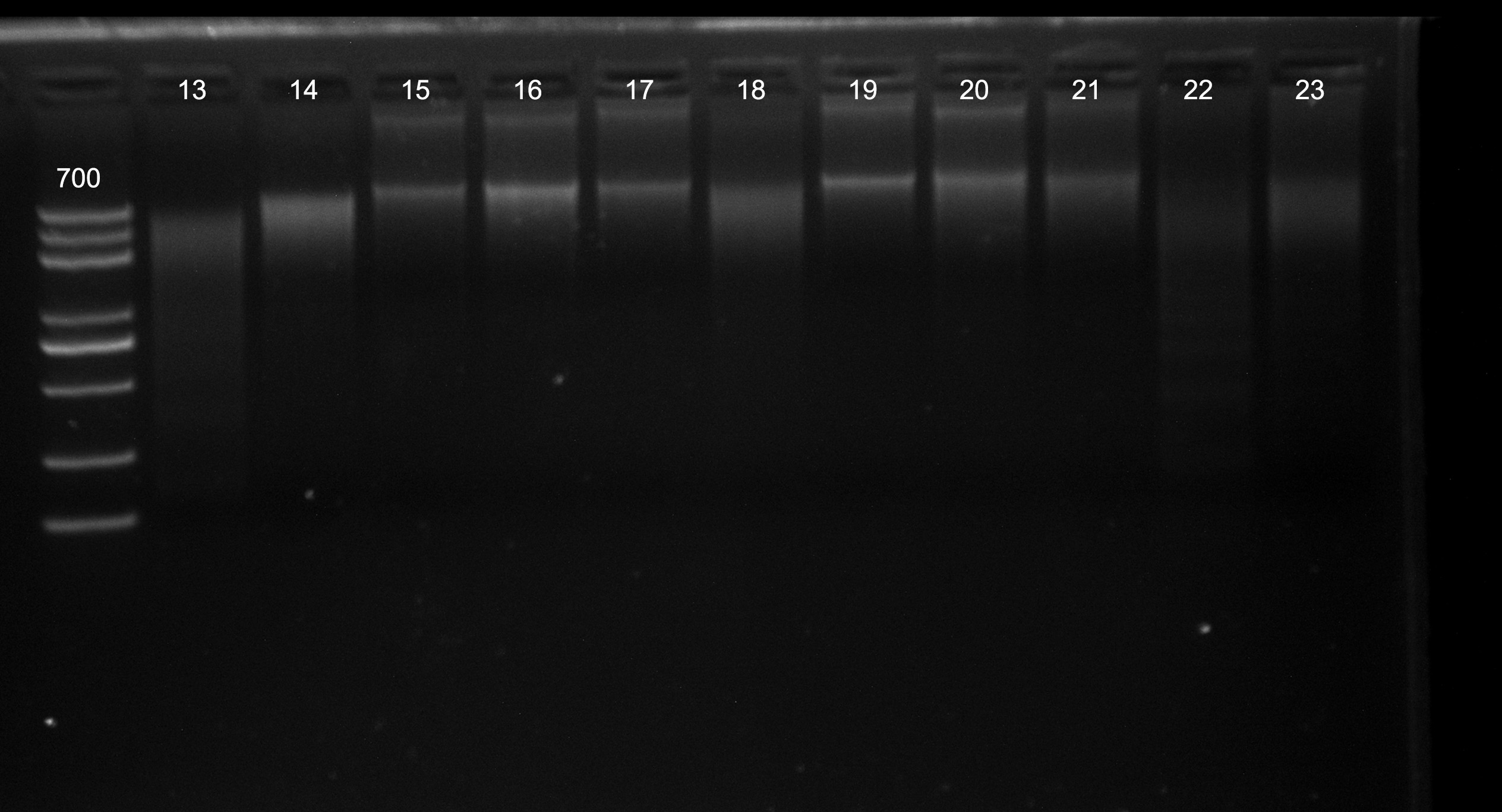

Supplement: Supplementary file 1 [file animals-16-01949-s001.zip › Figure S3.jpg]

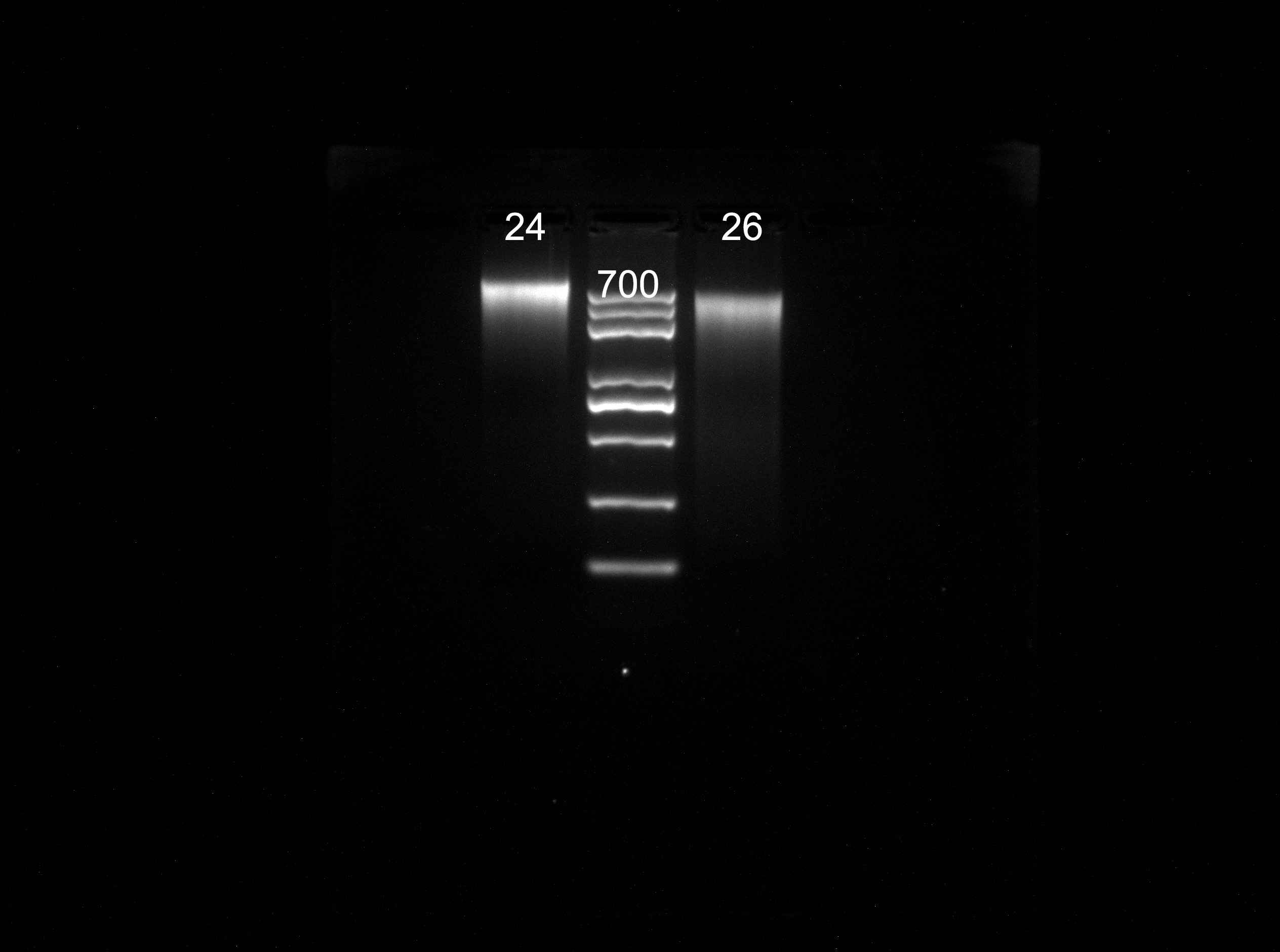

Supplement: Supplementary file 1 [file animals-16-01949-s001.zip › Figure S4.jpg]
